# Supplementary material for: Non-Structural Protein 2B of Human Rhinovirus 16 Activates Both PERK and ATF6 Rather Than IRE1 to Trigger ER Stress
Source: Viruses. 2019 Feb 1;11(2):133. doi: 10.3390/v11020133 (PMC6409610; doi:10.3390/v11020133)
Supplement: Supplementary file 1 [file viruses-11-00133-s001.pdf]

We extended time of virus infection from 9 h to 15 h post-infection or of p2B transfection from 36 h to 48 h (Figure S1 and S2). However, expression of p-IRE1 kept down-regulated by Western blotting and the XBP1s was not detected by RT-PCR. Thus, IRE1-XBP1 cannot function in ER stress from H1-HeLa cells infected HRV16.

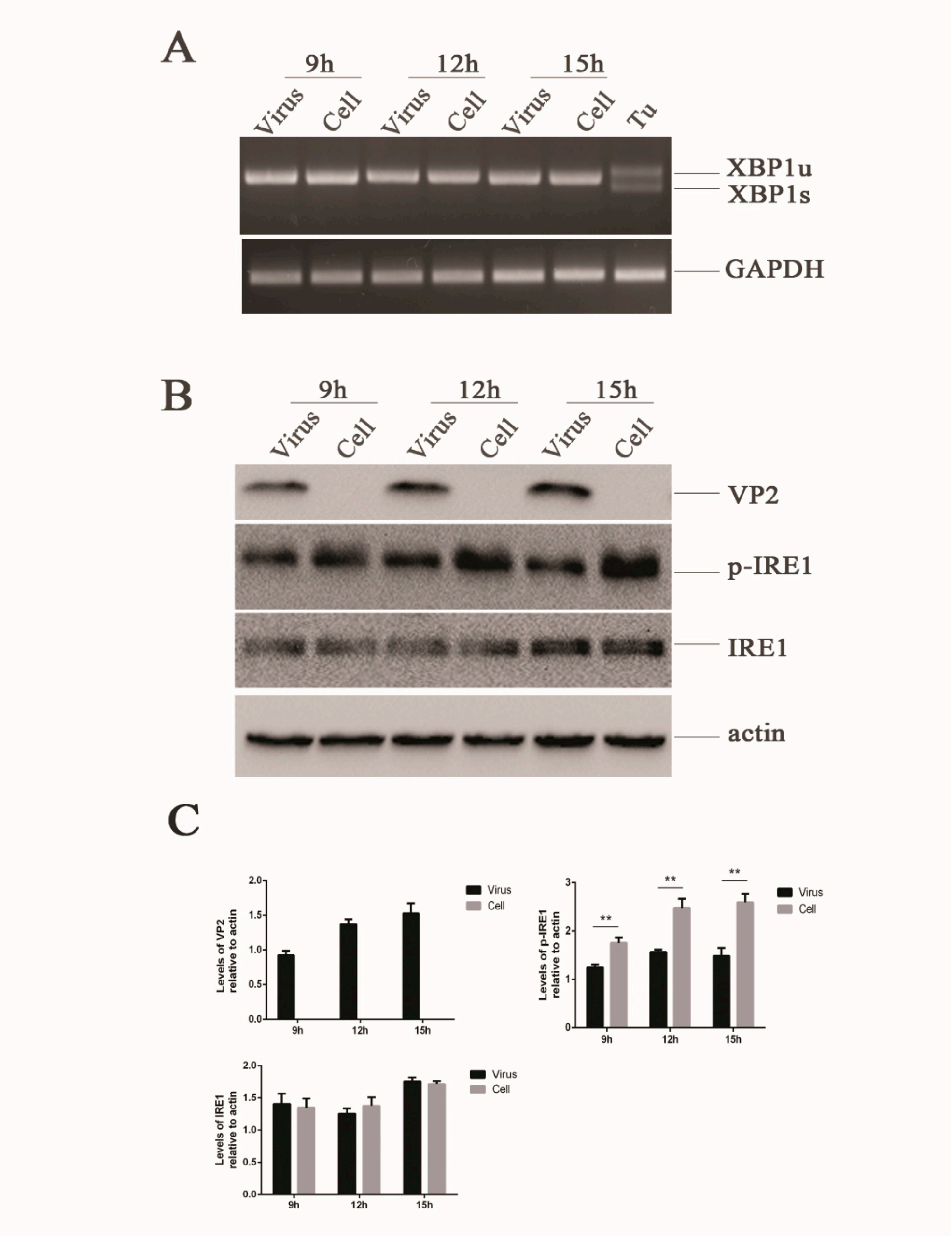

Figure S1 HRV16 infection induces dephosphorylation of IRE1. (A) Total RNA was extracted from cells after H1-HeLa cells were infected with HRV16 (MOI=5) for 9 h, 12 h and 15 h. The expression of XBP1 at the mRNA level was detected by RT-PCR. Cells treated with 1.0 µg/ml tunicamycin were used as a positive control. (B) The expressions of p-IRE1, IRE1 and actin were detected in H1-HeLa cells infected with HRV16 (MOI=5) for 9 h, 12 h and 15 h by Western blotting. Uninfected cells were used as controls. (C) Histogram of gray scanning analyses of the p-IRE1 and IRE1 protein bands of Figure 2B relative to actin was showed respectively. The error bars represent mean SD of three independent experiments. Statistical differences compared with controls were illustrated as * P<0.05, ** P<0.01, ***P<0.001.

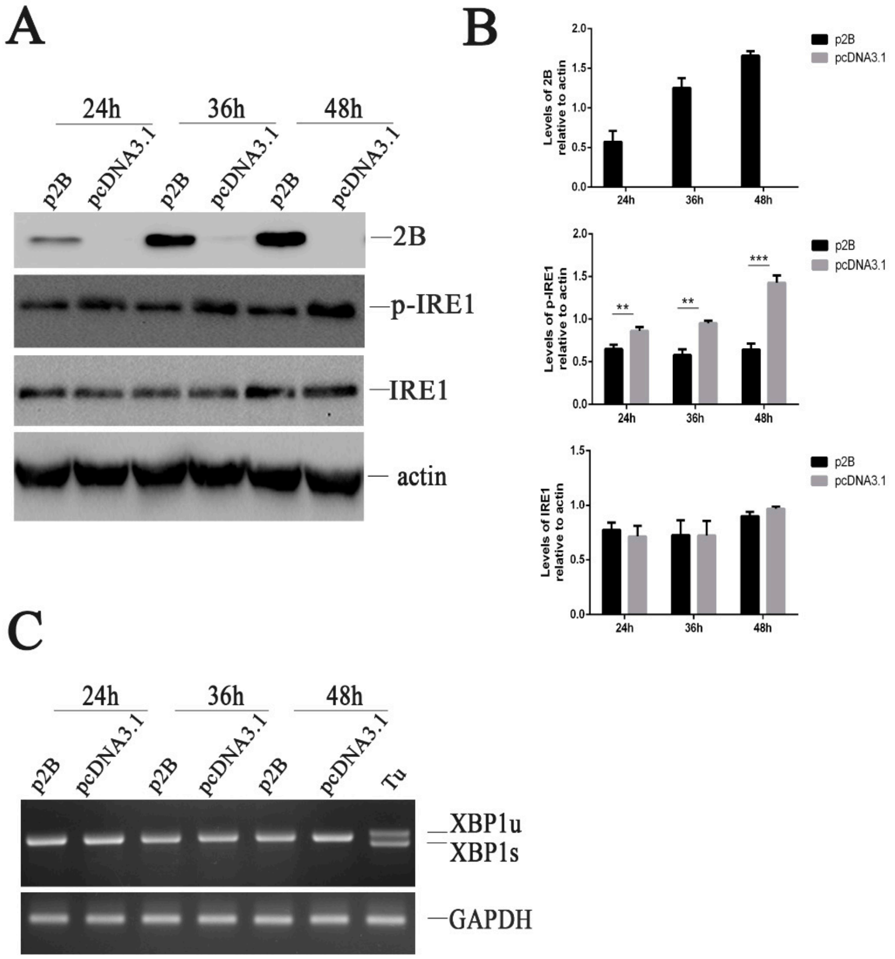

Figure S2 HRV16 2B dephosphorylates p-IRE1 protein. (A) H1-HeLa cells were transfected with p2B or pcDNA3.1 for 24 h, 36 h and 48 h. the expression of 2B, p-IRE1 and IRE1 protein was detected at each time point by Western blotting. β-actin was used as a control. (B) Histogram of gray scanning analyses of the 2B, p-IRE1 and IRE1 protein bands of Figure 5A relative to actin was showed, respectively. The error bars represent mean SD of three independent experiments. Statistical differences compared with controls were illustrated as * P<0.05, ** P<0.01, ***P<0.001. (C) H1-HeLa cells were transfected with p2B or pcDNA3.1 for 24 h, 36 h and 48 h. The time course of XBP1 mRNA expression was detected by RT-PCR. "Tu" represents cells treated with 1.0 μg/mL tunicamycin.
